# A bottom-up perspective on how fire changes ecosystem biogeochemistry via plant-soil interactions

**DOI:** 10.1007/s11104-025-08031-z

**Published:** 2025-11-19

**Authors:** Adam Pellegrini, Giacomo Certini, Minerva García-Carmona, Carmen Sánchez-García

**Affiliations:** 1 Department of Earth System Science, Doerr School of Sustainability, Stanford University, Stanford, CA 94305 USA; 2 Department of Agriculture, Food, Environment and Forestry, University of Florence, Florence, Italy; 3 Department of Agrochemistry and Environment, University of Miguel Hernández de Elche, Alicante, Spain; 4 Centre for Wildfire Research, Department of Geography, Swansea University, Swansea, UK; 5 European Commission, Joint Research Centre (JRC), Ispra, Italy

**Keywords:** Fire and biogeochemistry, Plant-soil interactions, Fire feedbacks, Soil organic matter, Microbial-plant interactions

## Abstract

**Background and Aims:**

The effect of fire on plants and soils cannot be viewed in isolation. Plant-soil interactions, and their role in determining the response of ecosystem to fire, has been a widely debated topic. Most studies describe patterns rather than the mechanisms that may lead to variable effects on soils across ecosystems.

**Methods:**

In this mini-review, we compile the literature on fire effects on soil processes to propose that a bottom-up framework considering plant-soil interactions is needed to explain the myriad of effects that fire has on soil biogeochemistry.

**Results:**

We highlight a number of processes that may be at play: (i) soil carbon saturation and mineral stabilization dynamics; (ii) nutrient-acquisition strategies (e.g., plant-microbial symbioses) and the emergence of biogeochemical feedbacks; (iii) physical soil changes that constrain carbon and nutrient turnover. We then highlight papers in this Special Issue on fire and plant-soil interactions that address these three processes to unpack how fire changes biogeochemical cycling in an ecosystem.

**Conclusion:**

We conclude that while shifts in plant biomass composition and inputs consistently influence soil properties across studies, increasing evidence shows the critical role of plant-soil interactions in determining belowground processes.

## Introduction

Wildfires are a natural ecological disturbance integral to the functioning of many ecosystems (Pausas and Keeley 2019). Fire-prone regions cover 70% of the total global stocks of topsoil organic carbon (Pellegrini et al. 2022), underscoring the importance of understanding how fire affects soil processes. A key reason for this understanding is that some effects can be long lasting; for example, the formation and stabilization of pyrogenic carbon that has accumulated and persisted in soils for millennia (Bird et al. 2015). At the same time, fire regimes are changing, with rising severity and frequency in many forests and declining frequency in many grasslands (Jones et al. 2024b).

Fire is not simply a combustive agent that emits carbon to the atmosphere or leaves charred materials behind in the soil, it is a thermal transformer of ecosystems (Pausas and Bond 2020). Fire’s effects unfold across multiple scales and timeframes, producing immediate impacts primarily driven by soil heating and long-term effects driven by ecological and geophysical changes, such as shifts in vegetation composition and biomass and loss in soil structure (Dunn and Debano 1977; Certini 2005). It is through these complex interactions that fire, both wild and prescribed, can change ecosystem biogeochemistry.

In this Review and Special Issue, we propose that understanding fire effects on soils requires considering plant-soil interactions in tandem. We are not referring to fire as the ‘top-down’ agent of change since it directly influences both aboveground processes (plant biomass amount and turnover) as well as belowground processes (thermal transformation of soils, combustion of organic matter and pyromineralization). Rather, our approach considers a ‘top-down’ perspective to be mostly focused on biomass inputs, and a ‘bottom-up’ perspective that considers microbially mediated processes and direct thermal impacts on physical changes in shaping plant-soil interactions. Each pathway should theoretically vary in its strength according to the amount of plant production and its responses to fire (i.e., greater role of top-down) vs. the physicochemical controls on decomposition and how it constrains carbon and nutrient cycling (i.e., greater role of bottom-up).

## Uncertainty in how fire affects soils

The effect of fire on plant-soil interactions has been a widely debated topic—with numerous global meta-analyses placing values on the changes in soil carbon and nutrient pools, microbial biomass and activity, and plant composition (Wan et al. 2001; Nave et al. 2011; Dooley and Treseder 2012; Pellegrini et al. 2018; Xu et al. 2022; Chai et al. 2025). However, these global synthesis studies overlook the mechanisms that may lead to variable effects on soils across ecosystems. For example, variation in microbial processing of organic matter can interact with soil minerals to influence soil carbon dynamics (Georgiou et al. 2022). Previous reviews have sought to integrate fire into a more holistic understanding of soils by evaluating its effects on specific pathways that alter carbon inputs, nutrient cycling, microbial activity and soil organic matter persistence (Certini 2005; Knicker 2007; Mataix-Solera et al. 2011). Yet few efforts have explicitly linked these mechanisms with broader global trends in how fire impacts carbon and nutrient storage and cycling in soils.

## The need for a bottom-up framework

**Fig. 1:**
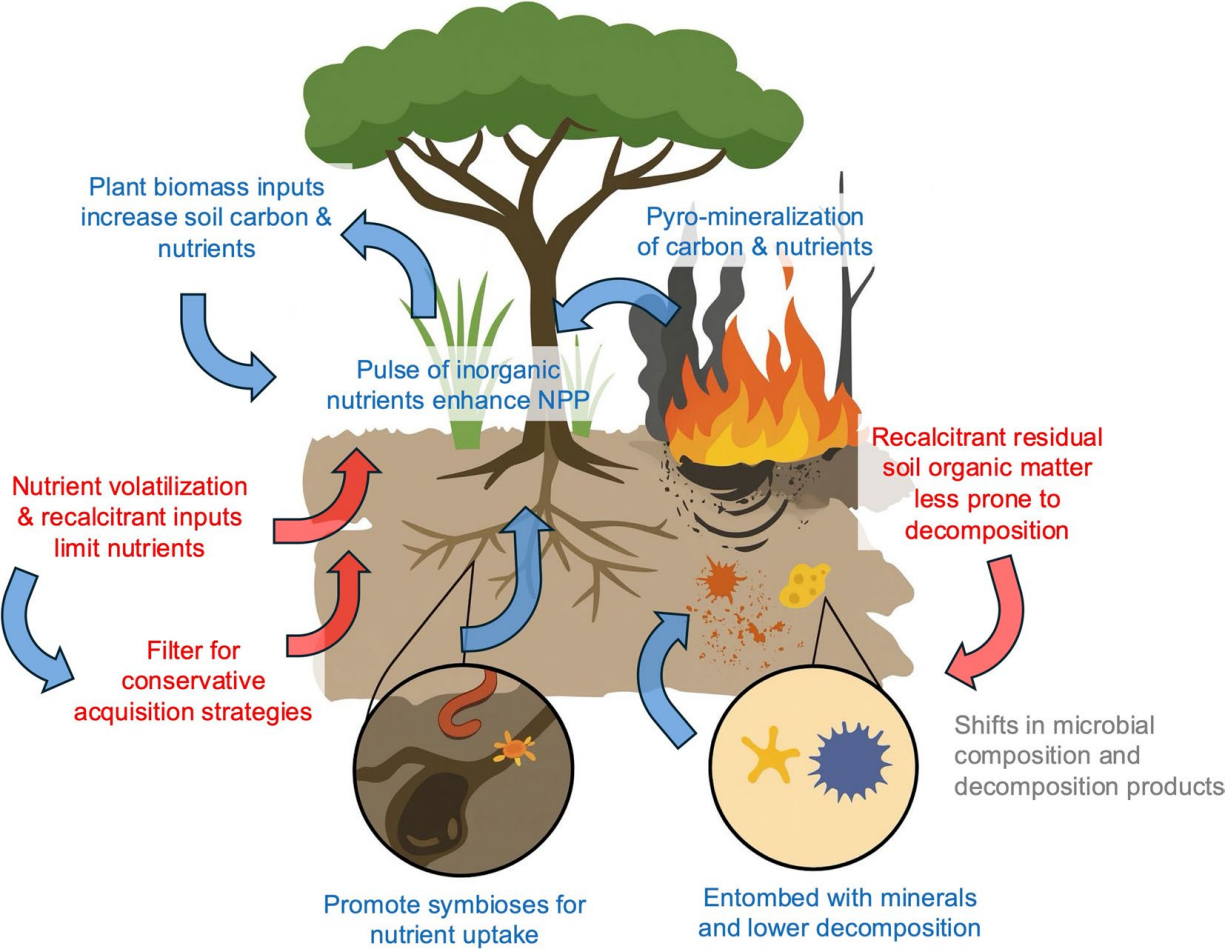
Conceptual framework for how bottom-up processes might regulate ecosystem responses to fire through plant-soil interactions. Colors illustrate the direction of the interaction, with blue representing positive interactions with plants and red being negative. The left-hand side describes plant-nutrient feedbacks, which are regulated by both the immediate pulse of inorganic nutrients and the longer-term changes in nutrients (especially declines in nitrogen). These can change the plant nutrient acquisition strategies to either lead to negative interactions with plant growth (conservative strategies such as ectomycorrhizal symbioses, long-lived roots and recalcitrant leaf litter) or positive interactions (acquisitive strategies such as nitrogen fixation, roots with rapid turner and nutrient-rich biomass). The right-hand side describes how microbial communities may interact with soil physicochemical properties to regulate the decomposition of organic matter and storage of soil carbon. Recalcitrant soil organic matter after fire (e.g., highly condensed aromatic compounds and, more generally, pyrogenic carbon) can slow decomposition and lead to greater storage of particulate forms. But shifts in microbial composition and decomposition that enhance growth efficiency can stimulate microbial turnover and associations with minerals

The dominance of a top-down framework for explaining fire effects on soils is especially apparent for soil organic carbon. In a top-down framework, shifts in soil carbon storage in fire-prone regions are hypothesized to be determined by biomass inputs resulting from changes in plant productivity and composition (Scurlock and Hall 1998; Smith 2014; Pellegrini et al. 2023b; Coetsee et al. 2023). However, under certain environmental conditions, changes in decomposition processes and rates and stabilization mechanisms may be equally important. For example, in soils with relatively high proportions of labile organic matter, increased microbial activity after fire may accelerate decomposition that could rapidly deplete carbon stocks. In contrast, in soils with low mineral saturation capacity, greater microbial turnover and efficiency could encourage the storage of more stable, mineral-associated organic carbon (Abramoff et al. 2021; Georgiou et al. 2025), thereby facilitating gains in soil carbon after fire. In the more extreme cases, burning soil organic matter in peat (i.e., ground fires) can have direct effects on microbial communities, transformation of soil organic matter that influences decomposition, and survival of plants (Allingham et al. 2024) (Fig. 1).

## Soil carbon stability and saturation

Soil carbon is the dominant terrestrial carbon storage pool on Earth and is especially important for determining the carbon balance of fire-prone ecosystems. In boreal forests and tundra, decomposition is constrained by cold climatic conditions leading to the accrual of thick organic horizons; while in savannas and grasslands, high allocation of plant biomass belowground implies that soils play a large role in ecosystem carbon dynamics (Jackson et al. 2017). As fire regimes shift due to climate change and land-use practices, the balance between carbon inputs and losses in these systems may be altered, with significant consequences for both regional and global carbon cycles (Andela et al. 2017; Pellegrini et al. 2018; Jones et al. 2024a).

Measuring soil carbon in constituent pools, such as mineral-associated organic carbon vs. free or aggregate-occluded particulate organic carbon, has improved our understanding of carbon accrual and stability in many contexts (Mastrolonardo et al. 2015; Cotrufo et al. 2019; Lavallee et al. 2020; Georgiou et al. 2022, 2024). Studies of fire effects on soils have also begun to account for these distinctions, but it remains unclear why fire changes the ‘stable’ soil carbon pool in some instances but not in others. For example, fire effects on aggregate stability have been a long-standing topic in fire science (Mataix-Solera et al. 2011), in many cases breaking down aggregates (Mastrolonardo et al. 2015). In contrast, the formation of pyrogenic oxides may act as a cementing agent, increasing aggregate stability (Jiménez-Pinilla et al. 2016).

Fire can also modify potential organo-mineral interactions. For one, it can change particle size—leading to finer particles and greater mineral surface area. Moreover, fire can alter the soil’s mineralogical assemblage. For example, at temperatures above 500 °C, kaolinite breaks down and goethite can transform into maghemite (Ketterings et al. 2000). Given that organic matter fractions associated with magnetic and non-magnetic minerals have been shown to differ in composition and turnover time (Chiti et al. 2019), these physical changes can directly modify the potential for organo-mineral associations. Yet, soil aggregate and/or organo-mineral association dynamics are rarely incorporated into fire models and global assessments of fire effects on soil carbon (Lasslop et al. 2020; Harrison et al. 2021). This reveals a disconnect between conceptual reviews and the quantitative assessment of fire effects on global biogeochemistry.

Pyrogenic carbon production is central to understanding how fire drives long-term stabilization of soil organic carbon, but its relative importance is biome-dependent and may be lower in ecosystems such as grasslands, where less char is produced (Santín et al. 2015, 2016; Jones et al. 2019). In savannas and grasslands, recent work has emphasized that the primary stabilization pathway is the association between organic matter and minerals (Georgiou et al. 2022; Bai and Cotrufo 2022). Fire can affect this pathway by shifting the relative inputs of particulate organic matter through changes in biomass turnover. Fire can also influence microbial transformations that contribute to the formation of mineral-associated organic matter by shifting plant-microbial interactions (Pellegrini et al. 2022). Additionally, post-fire conditions, such as increased solar radiation on the ground due to the absence of vegetation cover and the associated reduction in surface albedo, can further promote these transformations.

To better understand the fate of soil carbon following fire, it is important to examine not just carbon pools but also the underlying processes that regulate carbon turnover and stabilization. There are several factors relevant to fire, such as the quality of substrates entering the soil for decomposition (Hernández and Hobbie 2008; Holden et al. 2015). Advancing the field will require measuring fire effects on microbial carbon use efficiency, assessing their real impact on biological activity and biodiversity, separating soil organic matter into constituents more reflective of decomposability, and conducting experimental manipulations across soil type gradients.

## Plant-microbial symbioses

Plant–microbial interactions are increasingly recognized as central to understanding how ecosystems respond to fire (Hopkins and Bennet 2024; Zhou et al. 2024). Among microbes, fungi play fundamental roles, as their symbioses with plants regulate decomposition as well as soil carbon and nutrient cycling (Phillips et al. 2013; Zak et al. 2019). Certain fungal taxa even make remarkable contributions to soil carbon storage (Liang et al. 2019). While some studies link fire-induced changes in plant traits with decomposition and ecosystem-scale biogeochemical changes (Mack et al. 2021; Ibáñez et al. 2025), most fail to consider the nuances of microbial community shifts and their implications, or do so using correlational approaches (e.g., Pellegrini et al. 2021a).

Plant-microbial symbioses and rhizosphere interactions are likely important in fire-prone systems, particularly in ecosystems where root dynamics dominate belowground carbon inputs. For example, within savannas, root biomass allocation is hypothesized to contribute to biome stability (Walker et al. 1981), and root traits emerge as a promising pathway to predict the response of savanna trees to stress (Zhou et al. 2025). Moreover, fire can shift microbial community composition, e.g., decreasing the fungi to bacteria ratio (Pressler et al. forthcoming; Ibáñez et al. 2025) and favouring tree species that form symbioses primarily with ectomycorrhizal fungi (Jo et al. 2019; Pellegrini et al. 2021b). These are reciprocal dynamics, as changes in plant composition also influence microbial communities, with different plant strategies promoting different types of microbes (e.g., arbuscular mycorrhizae in soils (Pressler et al. forthcoming)). Part of the fungi-fire link may be driven by thermal tolerance (Glassman et al. 2016); however, whether ectomycorrhizal fungi are more or less sensitive to fire than other functional groups remains unclear (Taudière et al. 2017). Recent studies have also highlighted the importance of spore traits in predicting responses to fire (Hopkins et al. 2024). Thus, functional traits of both microbial and plant groups can help explain their coupled responses to fire.

## Root economic spectrum

Plant physiological characteristics are only one part of a species’ strategy for tolerating and recovering from fire. For example, thick bark can protect the cambium from overheating (Pausas 2015), low tissue N content and high N resorption can allow for a conservative N economy (Cavender-Bares and Reich 2012), and root biomass allocation can facilitate resource acquisition and rapid post-fire regrowth (Magaña-Hernández et al. 2020). More classical paradigms, such as resource limitation driving changes in plants with traits reflective of ‘fast’ vs. ‘slow’ life-history strategies, have been addressed in fire studies (Reich et al. 2001; Hoffmann et al. 2012; Pellegrini et al. 2023a). However, different categorizations, such as those based on root traits indicative of the reliance of plants on symbiotic fungi (Ma et al. 2018; Lu and Hedin 2019), might help predict belowground changes after fire.

Work focusing on resolving the responses of specific microbial groups, such as mycorrhizal fungi, is one path forward. For example, arbuscular mycorrhizal fungal lipid biomarkers can decrease linearly with longer fire return intervals (Pressler et al. forthcoming), suggesting that fire exclusion may inhibit resource acquisition by plants relying on arbuscular mycorrhizal fungi (e.g., grasses). In contrast, meta-analyses of tree community responses to fire find the opposite trend, with frequent burning filtering for tree species that associate with ectomycorrhizal fungi (Pellegrini et al. 2021b). A broad meta-analysis by Dove and Hart (2017) demonstrated that fire generally reduces mycorrhizal colonization, while recent work aims to improve the predictability of mycorrhizal fungi responses to fire using functional traits (Hopkins et al. 2024, 2025). Thus, these findings reveal no consistent trend in how fire filters plant strategies based on the root economic spectrum. Nonetheless, the importance of root traits for soil biogeochemical properties—such as the emergence of plant-soil feedbacks—points to the need for fire studies to incorporate root-microbe dynamics.

## Physical soil changes

Many of the prior examples of bottom-up processes rely on the indirect effects of fires on soils through shifts in the plant community, combined with alterations in microbial assemblages. However, fire has also direct effects on soils, some of which alter their physical properties, thereby shifting temperature and hydrological dynamics and, consequently, organic matter decomposition. Wildfire ash can also affect soil hydrology by, for example, increasing soil water retention, changing soil water repellency, or temporarily clogging soil pores (Stoof et al. 2010; Bodí et al. 2014; Kim et al. 2022). In ecosystems characterized by lower intensity fires, such as savannas and Mediterranean maquis, soils in plots where fire was excluded exhibited higher levels of hydrophobicity (Strydom et al. forthcoming; Capra et al. 2018).

Immediate physical effects via heating during a fire depend on fire intensity, soil moisture and soil type. Severe wildfires typically affect only the upper few centimetres of mineral soils (Debano 2000), while in organic soils they can penetrate much deeper (Walker et al. 2019). Even if soils do not heat to high levels, soil dwelling biota may still experience significant effects, ranging from temporary to long-lasting (Certini et al. 2021). Nevertheless, the impact of fire on soil physical properties—and the consequent effects on soil-dwelling biota—has not been fully disentangled and undoubtedly warrants further investigation.

Large direct effects are especially important in ground fires, where the soil itself is substantially burned. Ground fires tend to occur in soils rich in organic matter (Rein 2013) and can be intense when soils are dry enough for oxygen to allow for combustion to take place (Ward et al 2007). Peatlands are one representative ecosystem type that experiences ground fires (Turetsky et al 2015), and do not require high temperatures for the occurrence of large changes in organic matter. Physicochemical transformations in peat properties can influence microbial decomposition (Flanagan et al 2020; Leifeld et al 2018), which in turn influences both soil carbon stocks and nutrients available for regrowing plants. Taken together, bottom-up processes can also arise via direct effects of fire on soils.

## Special issue

In this special issue, papers collectively highlight the diversity of processes by which fire alters plant-soil-microbial interactions and the relative importance of these processes for predicting ecosystem responses.

Fire clearly impacts soil properties via top-down processes such as plant composition and productivity. For example, (Ibáñez et al. 2025) found that legume‐dominated patches initially had 25% higher soil carbon and 30% more total nitrogen pre‐burn, but 18 months after the fire, there were no more significant differences. Furthermore, Cai et al. (forthcoming) illustrated that post‐burn shifts in plant biodiversity favoured one species through increased resource capture and net positive biomass effects, whereas in another species the balance of trait and microbial changes yielded mixed indirect influences on biomass. In forests, tree species influenced soil formation processes such as podzolization (e.g., oaks had deeper E horizons) (Van Tran et al. 2025), but this effect interacted with bottom-up factors, as the deposition of pyrogenic carbon also affected soil by enhancing organic carbon in spodic horizons and increasing cation exchange capacity.

Changes in plant biomass inputs can have cascading effects on soil organic matter dynamics. Zhou et al. (2025) found that immediate fire-driven reductions in litterfall (−30%) partly explained declines in microbial necromass. However, 9 years post-fire, litter inputs had mostly recovered, supporting microbial necromass recovery and demonstrating the resilience of these processes over time.

Fire alters bottom-up processes by reducing microbial biomass, with ecosystems typically recovering over timescales of years to decades. For example, Ibáñez et al. (2025) found that i) microbial biomass under legumes declined by 40% immediately after burning but returned to 85% of pre‐burn levels within 18 months, and ii) phospholipid fatty acid markers showed decreased fungal:bacterial ratios immediately post‐burn, but these returned to pre-fire levels within 9 months. Another study found that fire reduced surface microbial necromass in the short‐term (1–5 yrs), while recovery, and even enhancement, occurred in the long‐term (> 9 yrs) (Zhou et al. 2025). These examples illustrate that microbial biomass is only part of the equation, as functional groups respond differently to fire, modifying community composition.

Shifts in soil biota composition and species interactions are also important bottom-up changes. In one case, soil fungal diversity rose by 18% after fire (Cai et al. forthcoming). Using a series of plots with varying fire return intervals, Pressler et al. (forthcoming) found that saprotrophic fungal biomarkers fell as fire return interval increased. Moreover, while total soil biota biomass remained stable regardless of fire return interval, community webs burned at a 4-yr interval lost complexity and stability due to reductions in fungal and predator groups, suggesting potential vulnerability under moderately infrequent fire regimes.

Fire-induced physical and biochemical alterations to soils are also important. In a forest ecosystem, charcoal retention enhanced organic carbon in spodic horizons and promoted horizon differentiation (Van Tran et al. 2025). In an African savanna, prescribed fire transiently impaired fine-pore conductivity without lasting effects, whereas prolonged fire exclusion accelerated macropore flow but hindered meso- and micro-pore infiltration (Strydom et al. forthcoming).

Top-down and bottom-up processes clearly interact, linking microbial dynamics with plant biomass responses. Cai et al. (forthcoming) found that while understory biomass remained unchanged, structural equation modelling showed an indirect positive relationship with fungal diversity. The link is especially apparent when investigating the response of symbiotic fungi, with arbuscular mycorrhizal fungal lipid biomarkers decreasing linearly with longer fire return intervals (Pressler et al. forthcoming). Consequently, fire exclusion may inhibit resource acquisition by plants relying on arbuscular mycorrhizal fungi. Other microbial-plant interactions can also change. For example, Hopkins and Bennet (2024) found that fire eliminated soil‐borne pathogens, neutralizing their pre‐burn negative feedback.

## Conclusion

The effects of fire on soils cannot be fully understood by focusing on aboveground changes in plants alone. While shifts in plant biomass composition and inputs consistently influence soil properties across studies, increasing evidence shows the critical role of belowground processes – including shifts in decomposer community and activity, soil physicochemical properties, and plant-microbial interactions. Testing new paradigms that consider how interactions between top-down and bottom-up processes drive changes in soil properties across temporal and spatial scales is an important future research direction in fire science.

## Data Availability

The manuscript uses no data.
